# Genome Analysis and Physiological Comparison of *Alicycliphilus denitrificans* Strains BC and K601^T^

**DOI:** 10.1371/journal.pone.0066971

**Published:** 2013-06-25

**Authors:** Margreet J. Oosterkamp, Teun Veuskens, Flávia Talarico Saia, Sander A. B. Weelink, Lynne A. Goodwin, Hajnalka E. Daligault, David C. Bruce, John C. Detter, Roxanne Tapia, Cliff S. Han, Miriam L. Land, Loren J. Hauser, Alette A. M. Langenhoff, Jan Gerritse, Willem J. H. van Berkel, Dietmar H. Pieper, Howard Junca, Hauke Smidt, Gosse Schraa, Mark Davids, Peter J. Schaap, Caroline M. Plugge, Alfons J. M. Stams

**Affiliations:** 1 Laboratory of Microbiology, Wageningen University, Wageningen, The Netherlands; 2 Los Alamos National Laboratory, Joint Genome Institute, Los Alamos, New Mexico, United States of America; 3 BioEnergy Science Center and Biosciences Division, Oak Ridge National Laboratory, Oak Ridge, Tennessee, United States of America; 4 Deltares, Utrecht, The Netherlands; 5 Laboratory of Biochemistry, Wageningen University, Wageningen, The Netherlands; 6 Microbial Interactions and Processes Research Group, Helmholz Centre for Infection Research, Braunschweig, Germany; 7 Research Group Microbial Ecology: Metabolism, Genomics and Evolution of Communities of Environmental Microorganisms, CorpoGen, Bogotá, Colombia; 8 Laboratory of Systems and Synthetic Biology, Wageningen University, Wageningen, The Netherlands; 9 Centre of Biological Engineering, University of Minho, Braga, Portugal; University of Groningen, The Netherlands

## Abstract

The genomes of the Betaproteobacteria *Alicycliphilus denitrificans* strains BC and K601^T^ have been sequenced to get insight into the physiology of the two strains. Strain BC degrades benzene with chlorate as electron acceptor. The cyclohexanol-degrading denitrifying strain K601^T^ is not able to use chlorate as electron acceptor, while strain BC cannot degrade cyclohexanol. The 16S rRNA sequences of strains BC and K601^T^ are identical and the fatty acid methyl ester patterns of the strains are similar. Basic Local Alignment Search Tool (BLAST) analysis of predicted open reading frames of both strains showed most hits with *Acidovorax* sp. JS42, a bacterium that degrades nitro-aromatics. The genomes include strain-specific plasmids (pAlide201 in strain K601^T^ and pAlide01 and pAlide02 in strain BC). Key genes of chlorate reduction in strain BC were located on a 120 kb megaplasmid (pAlide01), which was absent in strain K601^T^. Genes involved in cyclohexanol degradation were only found in strain K601^T^. Benzene and toluene are degraded via oxygenase-mediated pathways in both strains. Genes involved in the *meta-*cleavage pathway of catechol are present in the genomes of both strains. Strain BC also contains all genes of the *ortho*-cleavage pathway. The large number of mono- and dioxygenase genes in the genomes suggests that the two strains have a broader substrate range than known thus far.

## Introduction

Microbial (per)chlorate-reducing bacteria are able to produce oxygen as intermediate during anaerobic respiration with perchlorate and chlorate [1], [2], [3], [4]. This process can create possible advantages in *in-situ* bioremediation of anaerobic environments where pollutants like aromatic hydrocarbons persist that are more prone to aerobic degradation [1], [5]. *Alicycliphilus denitrificans* strain BC is able to couple benzene and toluene degradation to chlorate reduction [3]. Strain BC and *A. denitrificans* strain K601^T^ are members of the Comamonadaceae family of the Betaproteobacteria [3]. *A. denitrificans* strain K601^T^ was isolated with cyclohexanol and nitrate as substrates [6]. Contrary to strain BC, strain K601^T^ lacks the chlorate-reducing capability. Strain BC, on the other hand, cannot degrade cyclohexanol [3], [7].

Benzene degradation coupled to chlorate reduction was proposed to be an aerobic process, in which oxygen is derived from the conversion of chlorate is used in oxygenase-dependent pathways [3], [8], [9], [10]. Aerobic degradation of benzene and other aromatic hydrocarbons is well-studied [11], [12], [13]. In aerobic microorganisms, benzene degradation may be initiated by Rieske non-heme iron oxygenases, which catalyze a one-step incorporation of dioxygen into their substrates [14], [15] or by two successive monooxygenations. These monooxygenations are catalyzed by distinct multicomponent toluene/benzene monooxygenases, which produce intermediate phenols, and successively by multicomponent phenol monooxygenases, forming catechols [16]. The end product of catechol degradation is acetyl-CoA that can enter the citric acid cycle.

Degradation of aliphatic hydrocarbons, such as cyclohexanol and cyclohexanone can occur via aerobic and anaerobic pathways [17], [18], [19], [20]. Aerobic degradation of cyclohexanol is mediated by monooxygenases that cleave the aromatic ring. *A. denitrificans* strain K601^T^ degrades cyclohexanol under anaerobic conditions [6]. Likely, in the anaerobic cyclohexanol degradation pathway cyclohexanol is oxidized to 2-cyclohexenone via cyclohexanone. The enzyme that mediates conversion of 2-cyclohexenone is a bifunctional oxidoreductase that catalyzes both the Michael addition of water to 2-cyclohexenone and the subsequent oxidation of the resulting 3-hydroxycyclohexanone to 1,3-cyclohexanedione [7].

We aimed to obtain insight in the physiological properties of *A. denitrificans* strains BC and K601^T^ and in the pathways involved in degradation of aromatic and alicyclic compounds with different electron acceptors. Therefore, we studied the genome sequences of *A. denitrificans* strain BC and strain K601^T^ and performed more detailed physiological comparisons of these strains.

## Materials and Methods

### Cultivation and DNA Isolation

*A. denitrificans* strain BC (DSM 18852, JCM 14587) was isolated and described previously [3]. *A. denitrificans* strain K601^T^ (DSM 14773, CIP 107495) was purchased from the DSMZ, (Deutsche Sammlung von Mikroorganismen und Zellculturen GmbH, Braunschweig, Germany). The strains were cultivated in AW1-sulfate medium as described previously [21]. For DNA isolation cells were grown in 1.2L-bottles containing 500 mL medium with acetate (10 mM) and nitrate (10 mM). Cultures were incubated at 30°C without agitation. Cells were harvested by centrifugation and genomic DNA was isolated following the protocol for bacterial genomic DNA isolation using CTAB of DOE JGI (U.S. Department Of Energy, Joint Genome Institute, CA, USA). DNA concentration was measured using Nanodrop (Thermo scientific) and DNA integrity and quality were determined by loading the genomic DNA on a 1% agarose gel with size and concentration markers according to the instructions of DOE JGI.

### Growth Experiments

To determine the substrate spectrum of the *A. denitrificans* strains, different electron donors were tested in duplicate batches with nitrate (10 mM), oxygen (5% in headspace) or chlorate (10 mM) as electron acceptor. Late log-phase cells of strain BC grown on acetate (10 mM) and nitrate (10 mM) were used as inoculum (5%) for all batches except for batches with aromatic compounds as substrate. In these batches late log-phase cells grown on either benzene (repeated feeds of 0.5 mM) or acetate (10 mM) and chlorate (10 mM) were used as inoculum (5%–10%). Physiological properties of strain K601^T^ were described before [6], but additional substrate tests were performed. Late log-phase cells of strain K601^T^ grown on acetate (10 mM) and either oxygen (5% in headspace) or nitrate (10 mM) were used as inoculum in these tests. Growth was monitored by visual observation of turbidity and the decrease in electron acceptor and donor concentration. Analytical procedures were as described previously [3].

### Genome Sequencing, Assembly and Annotation

High molecular weight genomic DNA of *A. denitrificans* strains BC and K601^T^ was provided to the DOE JGI. For cloning and a combination of Illumina GAii and 454 shotgun sequencing [22], [23], a combination of small and large insert libraries were prepared. For strain BC the Illumina GAii shotgun library generated 32,476,780 reads comprising 1,169 Mb and for strain K601^T^ this generated 28,774,946 reads comprising 2,186 Mb. A 454 Titanium standard library generated 198,756 reads for strain BC and 637,992 reads for strain K601^T^. For strain BC a paired end 454 library generated 83,659 reads comprising 191 Mb and for strain K601^T^ this generated 314,193 reads comprising 281.7 Mb of 454 paired end data. All general aspects of library construction and sequencing performed at the JGI can be found at http://www.jgi.doe.gov/. The initial draft assembly of strain BC contained 120 contigs in 3 scaffolds and the draft assembly of strain K601^T^ contained 175 contigs in 2 scaffolds.

The 454 Titanium standard data and the 454 paired end data were assembled together with Newbler, version 2.3. The Newbler consensus sequences were computationally shredded into 2 kb overlapping fake reads (shreds). Illumina sequencing data were assembled with VELVET, version 0.7.63 [24], and the consensus sequences were computationally shredded into 1.5 kb shreds. We integrated the 454 Newbler consensus shreds, the Illumina VELVET consensus shreds and the read pairs in the 454 paired end library using parallel phrap, version SPS - 4.24 (High Performance Software, LLC). The software Consed was used in the following finishing process [25], [26], [27]. Illumina data were used to correct potential base errors and increase consensus quality using the software Polisher developed at JGI (www.jgi.doe.gov/software). Possible mis-assemblies were corrected using gapResolution (www.jgi.doe.gov/software), Dupfinisher [28], or sequencing cloned bridging PCR fragments with subcloning. Gaps between contigs were closed by editing in Consed, by PCR and by Bubble PCR primer walks (Cheng, unpublished). To close gaps and to raise the quality of the finished sequence, a total of 511 additional reactions were necessary for strain BC, for strain K601^T^ a total of 415 additional reactions were necessary. The total size of the genome of strain BC is 4,835,713 bp and the genome size of strain K601^T^ is 5,070,751 bp.

The final assembly is based on 191 Mb and 227 Mb of 454 draft data for strains BC and K601^T^, respectively. This provides an average 40× coverage for the genome of strain BC and an average 45× coverage of the genome of strain K601^T^. Additionally, the final genomes are based on 650 Mb and 2,099 Mb of Illumina draft data for strains BC and K601^T^, respectively, which provides an average 135× coverage of the genome of strain BC and an average 416.3× coverage of the genome of strain K601^T^.

Genes were identified using Prodigal [29] as part of the Oak Ridge National Laboratory genome annotation pipeline, followed by a round of manual curation using the JGI GenePRIMP pipeline [30]. The predicted CDSs were translated and used to search the National Center for Biotechnology Information (NCBI) nonredundant database, UniProt, TIGRFam, Pfam, PRIAM, KEGG, COG, and InterPro databases. These data sources were combined to assert a product description for each predicted protein. Non-coding genes and miscellaneous features were predicted using tRNAscan-SE [31], RNAMMer [32], Rfam [33], TMHMM [34], and signalP [35].

The complete final assemblies were released on September 3 2010 (strain BC) and on January 7 2011 (strain K601^T^). The genomes were implemented in GenBank. For strain BC the accession numbers are CP002449 (chromosome), CP002450 (megaplasmid), CP002451 (plasmid). For strain K601^T^ the accession numbers are CP002657 (chromosome) and CP002658 (plasmid).

### Bidirectional BLAST Analysis

The genomes of *A. denitrificans* strains BC and K601^T^ were compared using bidirectional BLAST analysis. The FTP server of NCBI (http://www.ncbi.nlm.nih.gov/Ftp) was used to download the protein sequence files obtained from the genome sequences of strains BC and K601^T^. Bidirectional best hits were obtained by BLAST using a similarity threshold of 50% and a sequence length mismatch of 80 to 120% [36].

### Comparative DNA and Cellular Property Analysis of Strains BC and K601^T^

The G+C content, DNA-DNA hybridization and cellular fatty acids analysis were performed by the DSMZ (Braunschweig, Germany). For cellular fatty acid analysis, strains BC and K601^T^ (DSM 14773^T^) were grown under identical conditions, i.e. acetate (10 mM) and nitrate (10 mM) in 2L AW-1-sulfate medium at 30°C [21]. ANI and TETRA of strain BC against strain K601^T^ and against *Acidovorax* sp. JS42, were determined using the software program JSpecies (http://www.imedea.uib.es/jspecies/). ANIb, ANIm and TETRA are calculated as described by Richter and Rosselló-Móra [37].

## Results and Discussion

### Comparison of Strains BC and K601^T^

The genomes of *A. denitrificans* strains BC and K601^T^ have been annotated [38]. Based on 16S rRNA gene sequence analysis *A. denitrificans* clusters in the family Comamonadaceae of the Betaproteobacteria [3]. Strains K601^T^ and BC showed 99.7% 16S rRNA gene similarity [3]. This study reveals that the three 16S rRNA gene copies present in each genome are identical for both strain BC and K601^T^, but have different gene location and orientation in each of the strains (Table S1), indicating that the genomes have a different topology. However, the general characteristics of the genomes of strains BC and K601^T^ are similar (Table 1). Furthermore, the strain-specific fatty acid methyl ester patterns of strains BC and K601^T^ are similar (Table S2).

**Table 1:** General features of the genomes of *A. denitrificans* strains BC and K601^T^.

		Strain BC	Strain K601^T^
Genome size		4,835,713 bp	5,070,751 bp
G+C content		67.9%	67.8%
DNA scaffolds		3	2
Chromosome	Size	4,637,013 bp	4,995,263 bp
	Coding DNA	91%	90%
	G+C content	68%	67%
Plasmid	Size	78,982 bp	75,488 bp
	Coding DNA	84%	87%
	G+C content	64%	62%
Megaplasmid	Size	119,718 bp	–
	Coding DNA	78%	–
	G+C content	58%	–
Total gene number		4709	4899
Protein coding genes		4542	4696
Pseudogenes rRNA genes		101	136
5S rRNA		3	3
16S rRNA		3	3
23S rRNA		3	3
tRNA genes		53	54

The G+C content of *A. denitrificans* strains K601^T^ and BC is 66.0 and 67.6 mol%, respectively, as determined by conventional methods [3], [6]. These values are comparable to those determined from the genomic DNA sequences of strains BC and K601^T^, which gave values of 67.9% and 67.8%, respectively. Experimental DNA-DNA hybridization of strain BC against K601^T^ showed 74.5±3.5% similarity. Based on the genome size difference this value seems low, but the genome of strain K601^T^ is about 7% larger than the genome of strain BC. Genome size differences may affect DNA-DNA hybridization values. However, the experimental error of DNA-DNA hybridization is too high for the genome size difference to have an effect. For species circumscription, a cut-off point of 70% DNA-DNA hybridization similarity is generally used. This cut-off point corresponds to 95% average nucleotide identity of genes present in both strains tested [39]. Based on this cut-off for average nucleotide identity, a tetranucleotide frequency correlation coefficient of >0.99 may support species delineation [37]. Values for average nucleotide identity (ANI) and the tetranucleotide frequency correlation coefficient (TETRA) can be determined using the software programme JSpecies [37]. According to JSpecies, the ANIb (BLAST calculation of ANI) value of strain BC compared to K601^T^ is 98.71% and the ANIm (MUMmer calculation of ANI) value is 99.60%, both well above the threshold of 95% for circumscribing species. The TETRA value was 0.9995, which is above the boundary of 0.99. In summary, ANIb, ANIm and TETRA values also indicate that both strains belong to the same species. As a comparison, we determined the values when comparing strain BC and another member of the Comamonadaceae family, *Acidovorax* sp. JS42, to which strain BC is closely related. In this case the ANIb value was 84.11%, the ANIm value 87.04% and the TETRA value 0.9756, confirming that these strains are different species, while there was 97% 16S rRNA similarity between the strains.

**Figure 1:**
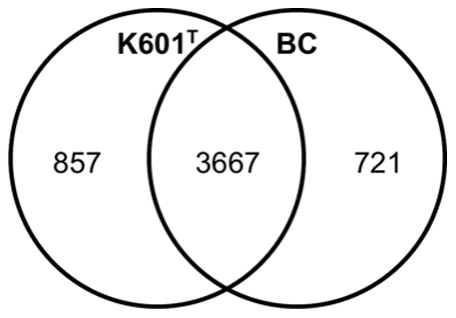
Bidirectional BLAST analysis of the genomes of *A. denitrificans* strains K601^T^ and BC. The amount of protein sequences present only in strains K601^T^ (left) and BC (right) and in both strains (center) is shown in the VENN diagram. 172 protein sequences of strain K601^T^ and 154 of strain BC could not be assigned (for instance duplicates of sequences).

**Figure 2:**
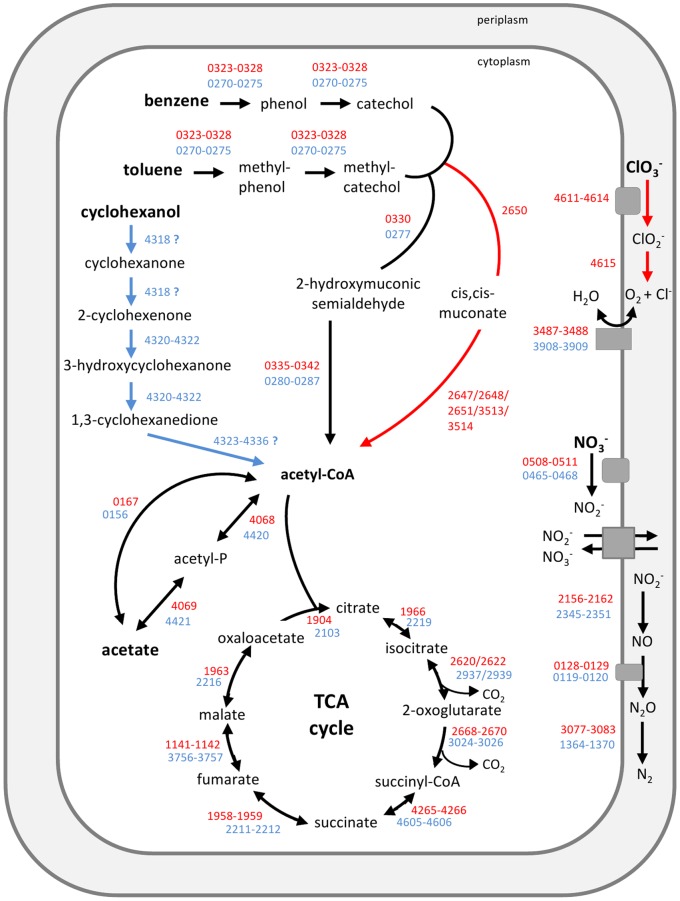
Main metabolic pathways of *A. denitrificans*. Pathways are indicated using arrows. Black arrows indicate pathways of both strain BC and K601^T^, red arrows indicate pathways of strain BC, and blue arrows pathways of strain K601^T^. Red gene numbers indicate genes of strain BC (geneID is Alide_red gene number) and blue gene numbers genes of strain K601^T^ (geneID is Alide2_blue gene number).

Bidirectional BLAST analysis showed that strain K601^T^ contains 857 proteins that are not present in strain BC and that strain BC has 721 proteins not present in strain K601^T^ (Fig. 1). An overview of the main metabolic pathways deduced from the genomes of the *A. denitrificans* strains is depicted in Fig. 2, and specific pathways for strain BC or K601^T^ are indicated. Lists of genes involved in these pathways are given from Table S3, S4, S5.

### Chlorate, Nitrate and Oxygen Respiration Pathways

In contrast to strain K601^T^, strain BC contains a megaplasmid harboring the genes involved in respiratory chlorate reduction (Alide01) [38]. To date, it is unknown if the megaplasmid can be transferred to other strains, e.g. to strain K601^T^ and if this plasmid allows other strains to grow by respiratory chlorate reduction.

**Figure 3:**
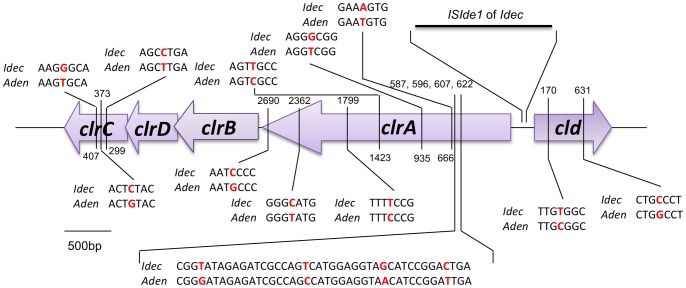
Gene cluster for chlorate reduction in *A. denitrificans* strain BC (*Aden*) compared to *I. dechloratans* (*Idec*). The gene cluster for chlorate reduction comprises of chlorite dismutase (*cld*), chlorate reductase subunit A, B, C and D (*clrA, clrB, clrC, clrD*), and in *I. dechloratans* it also includes an insertion sequence (*ISIde1*). The numbers represent the location of nucleotide differences (in red) of strain BC compared to *I. dechloratans* counted from the first nucleotide of each gene. The scale bar represents 500 bp. Sequences for the chlorate reduction gene cluster of *I. dechloratans* were obtained from the EMBL nucleotide sequence database (accession numbers AJ296077 and AJ566363).

Alide_4611–4614 encode subunits of a DMSO reductase family type II enzyme, or more specifically these genes encode chlorate reductase. Chlorate reductase is composed of four subunits encoded by the *clrABCD* genes (Alide_4611–4614). The chlorite dismutase gene (*cld*, Alide_4615) is transcribed in opposite direction (forward) compared to the genes encoding chlorate reductase (reverse). The gene cluster for chlorate reduction of strain BC is highly similar to the cluster of *Ideonella dechloratans* (Fig. 3), though it is not known whether the genes are plasmid-encoded in this bacterium [40], [41]. In *Dechloromonas aromatica* strain RCB genes encoding perchlorate reductase and chlorite dismutase are clustered and located on the chromosome (Daro_2580–2584, GenBank accession number of genome CP000089). These genes are clustered in *D. agitata* as well [42]. Recently, it was found that the genes encoding (per)chlorate reductase and chlorite dismutase in these *Dechloromonas* strains and two other (per)chlorate-reducing bacteria are located on a genomic island in the chromosomes [43].

Three types of nitrate reductases are known, the dissimilatory membrane-associated Nar, the dissimilatory periplasmic Nap and the assimilatory cytoplasmic Nas. Nas is exclusively involved in assimilatory nitrate reduction to ammonium [44], [45]. Nar and Nap differ with respect to chlorate reduction; Nar can catalyze chlorate reduction, but Nap cannot or only poorly reduce chlorate [46], [47], [48], [49]. Strains BC and K601^T^ have a functional Nar (the gene product of Alide_0508–0511 in BC and of Alide2_0465–0468 in K601^T^), but no functional Nap or Nas.

Putative nitrite reductase, nitric oxide reductase and nitrous oxide reductase encoding genes (*nir* genes: Alide_2156–2162 in BC and Alide2_2345–2351 in K601^T^, *nor* genes: Alide_0128 in BC and Alide2_0119 in K601^T^, and *nos* genes: Alide_3077–3083 in BC and Alide2_1364–1370 in K601^T^) indicate that nitrate is reduced to N_2_, which is in accordance with physiological tests [3], [6].

*A. denitrificans* strains BC and K601^T^ are facultative anaerobes [3], [6]. In the presence of oxygen, cytochrome oxidases catalyze the reduction oxygen to water, resulting in proton translocation and generating ATP by electron transport phosphorylation [50], [51]. Several cytochrome oxidases are known [52]. The genomes of strains BC and K601^T^ encode cytochrome oxidases that can be used under aerobic and micro-aerophilic (high-oxygen affinity) conditions. Cytochrome *c* oxidase genes are present (Alide_2815, 2816, 3608, 3609, 3487, 3488 in strain BC and Alide2_1643, 1644, 3908, 3909, 3953, 3954 in strain K601^T^). Furthermore, the genomes contain genes encoding cytochrome *o* ubiquinol oxidase (Alide_1992–1995 in strain BC and Alide2_2246–2249 in strain K601^T^) that are employed at high oxygen concentration. At low oxygen concentration, high-affinity cytochrome oxidases are used. The genomes of strains BC and K601^T^ contain cytochrome *bd* ubiquinol oxidase coding genes (Alide_2141 and 2142 in strain BC and Alide2_2330 and 2331 in strain K601^T^) and cytochrome *c* oxidase cbb3-type coding genes (Alide_3325–3328 in strain BC and Alide2_1119–1122 in strain K601^T^). An overview of genes involved in respiration in *A. denitrificans* strains BC and K601^T^ is shown in Table S4.

### Degradation Pathways of Aromatic and Alicyclic Compounds

**Figure 4:**
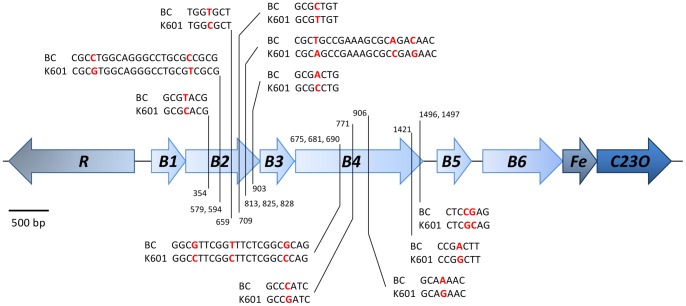
Organization of the multicomponent benzene/phenol monooxygenase cluster (B1–B6) and catechol dioxygenases (C23O) of *A. denitrificans* strains BC and K601^T^. In this gene cluster a gene coding for a transcriptional regulator (R) and a gene coding for a ferredoxin (Fe) were also found. Both strains BC and K601^T^ have highly similar gene clusters (99%) with differences only in subunit B2 and B4. The numbers represent the location of the nucleotide differences (in red) of strain BC compared to K601^T^ counted from the first nucleotide of each gene. The scale bar represents 500 bp.

Strains K601^T^ and BC are able to degrade benzene and toluene with oxygen, but not with nitrate as electron acceptor (Table 2), indicating that oxygenases are involved in the initial degradation steps of these aromatic compounds. In previous research, two oxygenase-coding genes involved in the initial successive oxidation reactions (*BC-BMOa*) and the subsequent cleavage of catechol (*BC-C23O*) were identified in the *Alicycliphilus* strains [3]. Monooxygenases that catalyze the conversion of benzene or toluene to phenol or methylphenol (benzene/toluene monooxygenases) and of phenols to catechols (phenol monooxygenases), belong to an evolutionary related family of soluble diiron monooxygenases [53]. Based on their alpha subunits, which are assumed to be the site of substrate hydroxylation, phenol as well as benzene/toluene monooxygenases can be differentiated within this family [53], [54]. Genome analysis confirmed the presence of a multicomponent phenol monooxygenase (Alide_0323–0328 in BC; Alide2_0270–0275 in K601^T^) [3]. The absence of other benzene/toluene mono- and/or dioxygenases suggests that the phenol monooxygenase is responsible for both the hydroxylation of benzene (and/or toluene) to (methyl-)phenol and the subsequent hydroxylation of (methyl-)phenol to (methyl-)catechol. Hydroxylation of the benzene ring catalyzed by phenol monooxygenases has been reported [16]. Phenol monooxygenase of *Pseudomonas stutzeri* strain OX1 transforms benzene and toluene to catechol and 3-methylcatechol (via phenol and 2-methylphenol), respectively [55]. Moreover, toluene-2-monooxygenase of *Burkholderia cepacia* strain G4 oxidizes toluene to 3-methylcatechol [56]. Although often lacking among phenol hydroxylase clusters [53], both strain BC and K601^T^ contain a gene coding for a ferredoxin (Alide_0329 in BC; Alide2_0276 in K601^T^) clustered with the phenol monooxygenase encoding genes. Furthermore, the phenol monooxygenase gene clusters contain genes encoding sigma54 specific transcriptional regulators (Alide_0322 and 0334 in BC; Alide2_0269 and 0279 in K601^T^) (Fig. 4). The protein products of Alide_0322 and Alide2_0269 have homology to regulatory proteins comprised in previously described phenol monooxygenase gene clusters, such as DmpR of *Pseudomonas sp.* strain CF600 (45% homology on protein level) that regulates transcription based on direct interaction with aromatic compounds [57]. A similar multicomponent phenol monooxygenase cluster is present in the close relative *Acidovorax sp.* strain JS42 (Ajs_0206–0210, which has 72% similarity on protein level) [58]. *D. aromatica* strain RCB and *Comamonas* sp. strain E6 contain similar monooxygenase clusters with 76% and 86% identity on protein level, respectively [59], and *D. aromatica* strain RCB also contains a benzene/toluene monooxygenase gene cluster.

**Table 2:** Overview of substrate range of *A. denitrificans* strains BC and K601^T^.

		Strain BC			Strain K601^T^	
Electrondonor	Concentration	NO_3_^−^	O_2_	ClO_3_^−^	NO_3_^−^	O_2_
Acetate	10 mM	+	+	+	+^a^	+^a^
Lactate	10 mM	+	+	+	+^a^	+^a^
Pyruvate	10 mM	+	+	+	+^a^	+^a^
Succinate	10 mM	+	+	+	+^a^	+^a^
Propionate	10 mM	+	+	+	+^a^	+^a^
Butyrate	10 mM	+	+	+	+^a^	+^a^
Malate	10 mM	+	+	+	+^a^	+^a^
Citrate	10 mM	+	+	+	+^a^	+^a^
Fumarate	10 mM	+	+	+	+^a^	+^a^
Glucose	10 mM	−	−	−	−a	−
Fructose	10 mM	−	−	−	−a	−
Xylose	10 mM	−	n.d.	n.d.	-a	n.d.
Alanine	10 mM	−	+	+	+	+
Glycine	10 mM	−	−	−	−	−
Glutamate	10 mM	+	+	+	+	+
Ethanol	10 mM	−	n.d.	n.d.	−a	−a
Methanol	10 mM	−	−	−	−a	−a
Glycerol	10 mM	−	n.d.	n.d.	n.d.	+^a^
Benzene	0.25 mM	−	+	+	−	+
Toluene	0.25 mM	−	+	+	−	+
Ethylbenzene	0.25 mM	−	−	−	−	−
*o*-Xylene	0.1 mM	−	−	−	−	−
*m*-Xylene	0.1 mM	−	−	−	−	−
*p*-Xylene	0.1 mM	−	−	−	−	−
Benzoate	1 mM	−	−	−	−a	−a
Phenol	1 mM	−	+	+	−a	+
*p*-Hydroxybenzoate	1 mM	−	−	−	−a	+^a^
*o*-Cresol	1 mM	−	+	+	−a	+^a^
*m*-Cresol	1 mM	−	+	+	−a	+^a^
*p*-Cresol	1 mM	−	+	+	−a	+^a^
Monochlorobenzene	0.05 mM	−	−	−	−	−
Catechol	1 mM	−	+	+	−	+
Cyclohexanol	1 mM	−	−	−	+^a^	+^a^

+: growth, −: no growth, n.d.: not determined,

a previous data [6].

Benzene and toluene degradation leads to the formation of (methyl)catechol. There are two routes of aerobic catechol degradation, the *meta*- and the *ortho*-cleavage pathway. All genes involved in the *meta*-cleavage pathway of (methyl)catechol degradation are present in the genomes of strains BC and K601^T^. We confirmed the presence of genes encoding a catechol 2,3-dioxygenase in strain BC as reported previously [3] and found homologous genes in strain K601^T^ (Alide_0330 in BC; Alide2_0277 in K601^T^). This catechol 2,3-dioxygenase catalyzes the extradiol cleavage of catechol to 2-hydroxymuconic semialdehyde. Among the 16 sequenced strains of Comamonadaceae only in five strains catechol 2,3-dioxygenase genes are present [54]. *Acidovorax sp*. JS42 contains a catechol 2,3-dioxygenase homologous to that of strains BC and K601^T^ with 92% similarity on protein level (Ajs_0214) [58]. Further degradation of 2-hydroxymuconic semialdehyde can proceed via the hydrolytic or the oxalocrotonate branch of the *meta*-cleavage pathway [60]. Genes encoding enzymes involved in both of these branches are present in the genomes of strains BC and K601^T^ (Fig. 2). The hydrolytic branch is used when toluene is converted via 3-methylcatechol and involves degradation of 2-hydroxymuconic semialdehyde to 2-oxopent-4-enoate. The enzyme catalyzing this conversion is a 2-hydroxymuconic semialdehyde hydrolase (Alide_0336 in BC; Alide2_0281 in K601^T^). Methyl-catechol and catechol are converted to 2-oxopent-4-enoate using the oxalocrotonate branch of the meta-cleavage pathway, which proceeds via a dehydrogenase, tautomerase and decarboxylase (Alide_0335+0340+0342 in BC; Alide2_0280+0285+0287 in K601^T^). Finally, 2-oxopent-4-enoate is converted to acetyl-CoA that can enter the citric acid cycle and the genes encoding the enzymes involved in this conversion are Alide_337–339 in strain BC and Alide2_282–284 in strain K601^T^.

Strain BC, but not K601^T^, also contains all genes essential for *ortho*-cleavage of catechol. In this pathway, catechol is converted by catechol 1,2-dioxygenase (encoded by Alide_2650) to *cis,cis*-muconate, which via muconolactone, 3-oxoadipate-enol-lactone, 3-oxoadipate and 3-oxoadipyl-coA, is converted to acetyl-coA (Alide_2647+2648+2651+3513+3514 of strain BC) [61]. Strain K601^T^ lacks genes coding for catechol 1,2-dioxygenase, muconate cycloisomerase and 3-oxoadipate-enol-lactonase, rendering the *ortho*-cleavage pathway incomplete.

**Figure 5:**
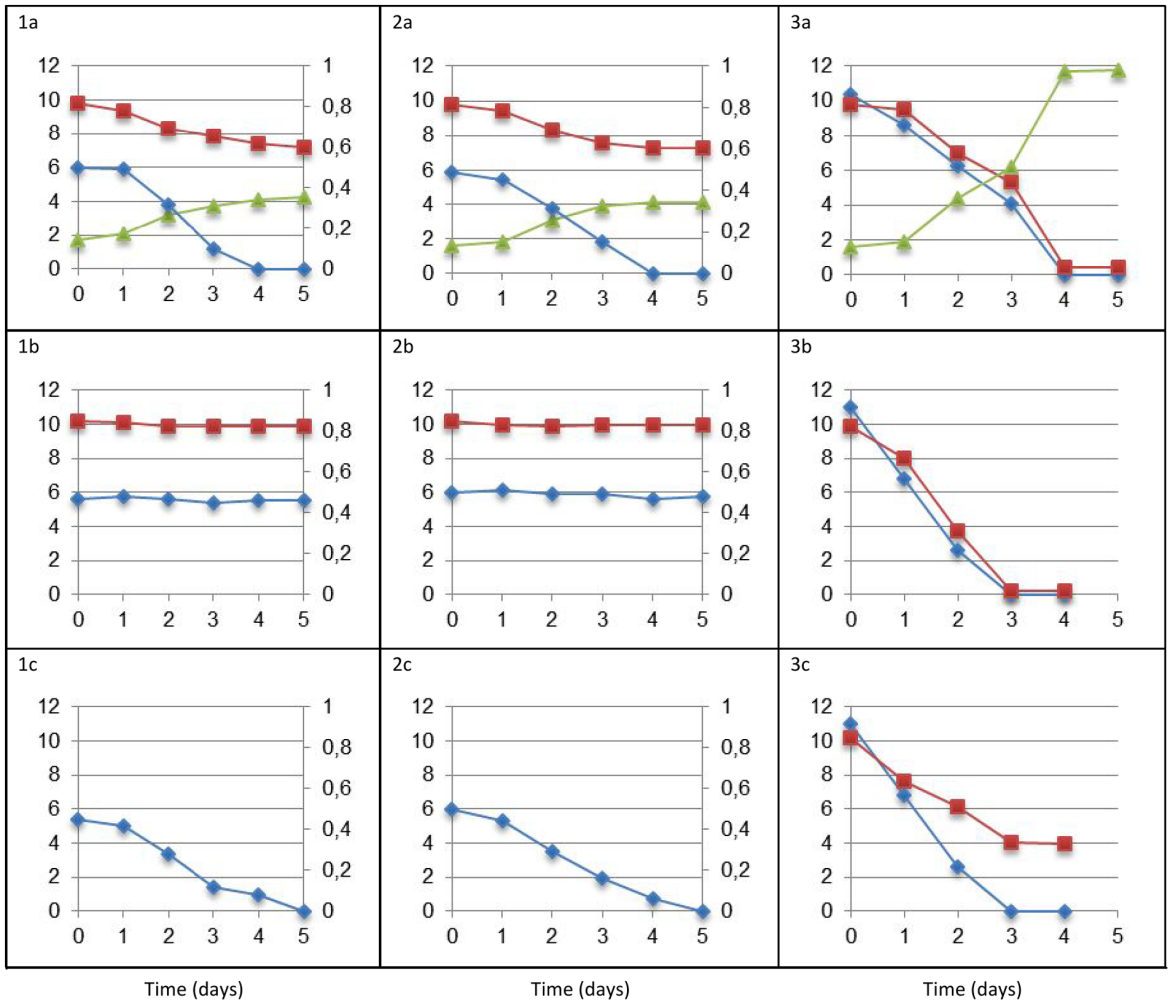
Degradation of benzene (1), toluene (2) and acetate (3) with chlorate (a), nitrate (b) or oxygen (c) as electron acceptor by *A. denitrificans* strain BC. Benzene, toluene and acetate degradation is indicated with diamonds. Benzene and toluene concentrations are outlined on a secondary y-axis while acetate and electron acceptor concentrations are indicated on the primary y-axis. Chlorate, nitrate and oxygen consumption is depicted with squares. Chloride production when chlorate is used as electron acceptor is indicated with triangles and no electron acceptor consumption is shown when no significant difference could be observed because of presence of the electron acceptor in abundance.

Although anaerobic benzene degradation was described for some pure bacterial cultures, information about the degradation pathways is incomplete [62], [63], [64], [65]. *D. aromatica* strain RCB is capable of anaerobic degradation of all BTEX compounds with nitrate as electron acceptor, but *A. denitrificans* strain BC and K601^T^ cannot degrade these compounds with nitrate while acetate can be degraded with nitrate as electron acceptor (Fig. 5). This is confirmed by the absence of genes that code for known key enzymes for anaerobic aromatic degradation in the genomes of strain BC and K601^T^, such as benzylsuccinate synthase or ethylbenzene dehydrogenase. Remarkably, these key enzymes are also not present in *D. aromatica* strain RCB [59]. Strain RCB is able to couple benzene degradation to nitrate reduction, but the occurrence of a strict anaerobic pathway is not proven, and it has been suggested that strain RCB might activate benzene with oxygen produced from the reduction of nitrate or uses hydroxyl free radicals [63], [66]. Oxygen production by nitrite reduction was found in the anaerobic methane degrading *Candidatus Methylomirabilis oxyfera* [67], [68] and was reported as a possible mechanism for the initial alkane activation in strain HdN1 [69]. A similar mechanism was proposed for benzene and toluene degradation using chlorate as electron acceptor in strain BC [3]. Physiological tests showed that these compounds are degraded in 3 to 5 days in presence of chlorate or oxygen (Fig. 5). Both strains BC and K601^T^ are unable to aerobically utilize ethylbenzene, xylenes and benzoate (Table 2). Accordingly, enzymes involved in the initial steps of degradation of these compounds are not present in the genomes.

Cyclohexanol degradation by strain K601^T^ follows a proposed pathway via the intermediates cyclohexanone, 2-cyclohexenone, 3-hydoxycyclohexanone to 1,3-cyclohexanedione and subsequent cleavage of the ring [7]. Genes encoding a three subunit molybdoenzyme (Alide2_4320– Alide2_4322) are present in the genome of strain K601^T^, but are absent in strain BC. This gene cluster, homologous to carbon monoxide dehydrogenase (CO-DH) and xanthine dehydrogenase (Xdh), is identified as a bifunctional hydratase/alcohol dehydrogenase (MhyADH), belonging to the molybdopterin binding oxidoreductase family with the double function of hydration of cyclohexenone and oxidation of its product 3-hydroxycyclohexanone to 1,3-cyclohexanedione [7]. An ORF located close to the MhyADH shows a sequence (Alide2_4318) with similarity to a flavin-containing domain of fumarate reductase/succinate dehydrogenase and 3-ketosteroid dehydrogenase (KSTD). Based on the reactions catalyzed by those types of enzymes, this sequence might be coding for a cyclohexanone dehydrogenase [7]. Depending on the substrate specificity of the coding enzyme, the conversion of cyclohexanol to cyclohexanone could also be catalyzed by this enzyme. Since members of this group of enzymes are known to catalyze the oxidative hydroxylation of a wide range of aldehydes and aromatic heterocyclic compounds [70] the substrate range of strain K601^T^ might be broader than known so far. More genes closely located to the MhyADH cluster are identified as CO-DH genes (Alide2_4319; Alide2_4326) and are possibly also involved in cyclohexanol degradation.

A cyclopentanol dehydrogenase (Alide2_4312) and, furthermore, more mono- and dioxygenase genes were found in the genomes than the ones we already described, such as an extradiol ring cleavage dioxygenase (Alide_2035 in BC; Alide2_2289 in K601^T^), a cytochrome P450 (Alide_3136 in BC; Alide2_1311) and several 2-nitropropane dioxygenases (Alide_0303, Alide_0687, Alide_2358, Alide_3754, Alide_3890, Alide_4340 in strain BC; Alide2_0250, Alide2_1856, Alide2_2552, Alide2_4108, Alide2_4236, Alide2_4670 in K601^T^). This indicates that a broader spectrum of xenobiotic compounds might be degraded by the *Alicycliphilus* strains. A list of genes involved in degradation of aromatic and alicyclic compounds in strains BC and K601^T^ can be found in Table S5.

### Other Physiological Characteristics

Strains BC and K601^T^ were physiologically characterized previously [3], [6]. Additional physiological tests were performed based on the genome sequences. Genome analysis showed that there are no known sugar transporter genes in strains BC and K601^T^. We did not observe growth of strains BC and K601^T^ with glucose and fructose (Table 2), though previously strain K601^T^ was described to use these sugars [6]. Comparative genome analysis showed that all genes of the tricarboxylic acid cycle are present in strains BC and K601^T^ (Table S3). The two bacteria use carboxylic acids like acetate, lactate, succinate or fumarate as substrates for growth (Table 2). Strains BC and K601^T^ can use the amino acids glutamate and alanine as growth substrates and genes encoding glutamate dehydrogenase (Alide_0201+1063 in BC; Alide2_0190+4027 in K601^T^) and beta-alanine-pyruvate transaminase (Alide_4363 in BC; Alide2_4693 in K601^T^) were found in the genome.

### Concluding Remarks

Bacteria that degrade benzene and other aromatic hydrocarbons in the absence of oxygen have two strategies for degradation: 1) employment of alternative pathways which are oxygenase-independent [4], [62], [71], [72] and 2) as described here, production of oxygen in the reduction of the electron acceptor to employ oxygenase-dependent pathways. Here we present genome information that shows how *A. denitrificans* strain BC is able to couple benzene degradation to chlorate reduction. The key genes that code for enzymes that are essential for chlorate reduction and oxygen production are located at a plasmid. *A. denitrificans* strain K601^T^ lacks this plasmid and thus is not able to degrade benzene with chlorate. The two strains are not able to degrade benzene with nitrate. Only a few pure cultures of anaerobic benzene-degrading bacteria have been described. *Azoarcus* strain DN11 and AN9, *Dechloromonas aromatica* strain RCB and JJ and a *Bacillus cereus* strain were reported to degrade benzene with nitrate as electron acceptor [66], [73], [74], [75]. Recently, *Bacillus subtilis* and *Pseudomonas aeruginosa* strains were found to degrade benzene with nitrate and oxygen as electron acceptors [76]. Thus far, it is not clear how these bacteria degrade benzene in the absence of oxygen. One of the options is an aerobic pathway involving oxygen derived from nitrate.

As aromatic hydrocarbons often accumulate in the anaerobic zones of soil, bacteria that are able to degrade hydrocarbons in the absence of oxygen are important for *in situ* bioremediation. However, oxygen is often difficult to introduce in soil. Chlorate and nitrate addition is an alternative to stimulate the breakdown of aromatic and aliphatic hydrocarbons. *A. denitrificans* strain BC has the ability to degrade some aromatic hydrocarbons, but its substrate range is limited. However, the observation that the essential genes for chlorate reduction (chlorate reductase and chlorite dismutase) are coded on a plasmid suggests that the ability to degrade hydrocarbons with chlorate can be transferred to bacteria with a wider substrate spectrum such as e.g. *Pseudomonas putida*. An important prerequisite, however, may be that the oxygenases possess a high affinity for oxygen.

## Supporting Information

Table S1 Location of the 16S and 23S rRNA genes in the genomes of *A. denitrificans* strains BC and K601^T^.
(DOCX)

Table S2 Major cellular fatty acids of *A. denitrificans* strains BC and K601^T^. Data of a previous study of strain K601^T^ [6] are included.
(DOCX)

Table S3 List of genes involved in the citric acid cycle and acetate metabolism in *A. denitrificans* strains BC and K601^T^.
(DOCX)

Table S4 List of genes involved in anaerobic respiration in *A. denitrificans* strains BC and K601^T^.
(DOCX)

Table S5 List of genes involved in degradation of aromatic and acyclic compounds in *A. denitrificans* strains BC and K601^T^.
(DOCX)
