# Supplementary material for: Genome Analysis and Physiological Comparison of Alicycliphilus denitrificans Strains BC and K601T
Source: PLoS One. 2013 Jun 25;8(6):e66971. doi: 10.1371/journal.pone.0066971 (PMC3692508; doi:10.1371/journal.pone.0066971)
Supplement: Table S1 — Location of the 16S and 23S rRNA genes in the genomes of A. denitrificans strains BC and K601T. (DOCX) [file pone.0066971.s001.docx]

|  | | **Location in strain BC** | | | **Location in strain K601^T^** | | |
| --- | --- | --- | --- | --- | --- | --- | --- |
| **Gene** | | 1 | 2 | 3 | 1 | 2 | 3 |
| **16S rRNA** | Start (nucl) | 3143946 | 4003821 | 4282912 | 1500507 | 4372093 | 4663323 |
|  | Stop (nucl) | 3142430 | 4002305 | 4281396 | 1502023 | 4370577 | 4661807 |
|  | Length (bp) | 1517 | 1517 | 1517 | 1517 | 1517 | 1517 |
|  | Orientation | - | - | - | + | - | - |
| **23S rRNA** | Start (nucl) | 3141781 | 4001656 | 4280747 | 1502672 | 4369928 | 4661158 |
|  | Stop (nucl) | 3138905 | 3998780 | 4277871 | 1505548 | 4367051 | 4658282 |
|  | Length (bp) | 2877 | 2877 | 2877 | 2877 | 2878 | 2877 |
|  | Orientation | - | - | - | + | - | - |
