# Supplementary material for: Genome Analysis and Physiological Comparison of Alicycliphilus denitrificans Strains BC and K601T
Source: PLoS One. 2013 Jun 25;8(6):e66971. doi: 10.1371/journal.pone.0066971 (PMC3692508; doi:10.1371/journal.pone.0066971)
Supplement: Table S2 — Major cellular fatty acids of A. denitrificans strains BC and K601T. Data of a previous study of strain K601T [6] are included. (DOCX) [file pone.0066971.s002.docx]

|  | **Strain BC** | **Strain K601^T^** | **Strain K601^T^** |
| --- | --- | --- | --- |
|  | **(this study)** | **(this study)** | **(previous study)** |
| **Major cellular fatty acids** | **(%)** | **(%)** | **(%)** |
| C10:0 | 0.4 | 0.6 | - |
| C10:0 3OH | 2.4 | 4.2 | -^a^ |
| C12:0 | 2.7 | 4.3 | 4 |
| C14:0 | 1.7 | 1.4 | - |
| C15:0 | n.d. | n.d. | 2 |
| C16:1 ω7c | 10.9 | 27.0 | 37 |
| C16:0 | 44.0 | 36.2 | 24 |
| C17:1 ω7c | 0.3 | n.d | - |
| cycloC17:0 | 27.8 | 9.9 | 2 |
| C18:3 ω6c (6,9,12) | n.d. | 0.4 | - |
| C18:1 ω7c | 8.4 | 13.6 | 21 |
| C18:0 | 0.25 | 0.4 | - |
| cycloC19:0 ω8c | 1.25 | 2.1 | - |

n.d.: not detectable, -: unknown and ^a^: occurred in ‘small amounts’.
