# Supplementary material for: Genome Analysis and Physiological Comparison of Alicycliphilus denitrificans Strains BC and K601T
Source: PLoS One. 2013 Jun 25;8(6):e66971. doi: 10.1371/journal.pone.0066971 (PMC3692508; doi:10.1371/journal.pone.0066971)
Supplement: Table S3 — List of genes involved in the citric acid cycle and acetate metabolism in A. denitrificans strains BC and K601T. (DOCX) [file pone.0066971.s003.docx]

| **Enzyme name** | **Reaction** | **GeneID in BC** | **GeneID in K601^T^** |
| --- | --- | --- | --- |
| Aconitase | citrate : isocitrate | Alide_1966 | Alide2_2219 |
| Isocitrate dehydrogenase | isocitrate : 2-oxoglutarate | Alide_2620 | Alide2_2937 |
|  |  | Alide_2622 | Alide2_2939 |
| 2-oxoglutarate dehydrogenase | 2-oxoglutarate : succinyl-CoA | Alide_2668 | Alide2_3024 |
|  |  | Alide_2669 | Alide2_3025 |
|  |  | Alide_2670 | Alide2_3026 |
| Succinyl-CoA synthetase | succinyl-CoA : succinate | Alide_4265 | Alide2_4605 |
|  |  | Alide_4266 | Alide2_4606 |
| Succinate dehydrogenase | succinate : fumarate | Alide_1958 | Alide2_2211 |
|  |  | Alide_1959 | Alide2_2212 |
| Fumarate hydratase | fumarate : malate | Alide_1141 | Alide2_3756 |
|  |  | Alide_1142 | Alide2_3757 |
| Malate dehydrogenase | malate : oxaloacetate | Alide_1963 | Alide2_2216 |
| Citrate synthase | oxaloacetate : citrate | Alide_1904 | Alide2_2103 |
| Acetate kinase | acetate : acetyl-phosphate | Alide_4069 | Alide2_4421 |
| Phosphate-acetyltransferase | acetyl-phosphate : acetyl-CoA | Alide_4068 | Alide2_4420 |
| Acetyl-CoA hydrolase/transferase | acetate : acetyl-CoA | Alide_0167 | Alide2_0156 |
| Acetate/CoA ligase | acetate : acetyl-CoA | Alide_1147 | Alide2_3751 |
