# Supplementary material for: Genome Analysis and Physiological Comparison of Alicycliphilus denitrificans Strains BC and K601T
Source: PLoS One. 2013 Jun 25;8(6):e66971. doi: 10.1371/journal.pone.0066971 (PMC3692508; doi:10.1371/journal.pone.0066971)
Supplement: Table S4 — List of genes involved in anaerobic respiration in A. denitrificans strains BC and K601T. (DOCX) [file pone.0066971.s004.docx]

| **Enzyme name** | **Reaction** | **GeneID in BC** | **GeneID in K601^T^** |
| --- | --- | --- | --- |
| Chlorate reductase | Chlorate : chlorite | Alide_4611 | - |
|  |  | Alide_4612 | - |
|  |  | Alide_4613 | - |
|  |  | Alide_4614 | - |
| Chlorite dismutase | Chlorite : chloride | Alide_4615 | - |
| Nitrate reductase | Nitrate : nitrite | Alide_0508 | Alide2_0465 |
|  |  | Alide_0509 | Alide2_0466 |
|  |  | Alide_0510 | Alide2_0467 |
|  |  | Alide_0511 | Alide2_0468 |
| Nitrite reductase | Nitrite : nitric oxide | Alide_2156 | Alide2_2345 |
|  |  | Alide_2157 | Alide2_2346 |
|  |  | Alide_2158 | Alide2_2347 |
|  |  | Alide_2159 | Alide2_2348 |
|  |  | Alide_2160 | Alide2_2349 |
|  |  | Alide_2161 | Alide2_2350 |
|  |  | Alide_2162 | Alide2_2351 |
| Nitric oxide reductase | Nitric oxide : nitrous oxide | Alide_0128 | Alide2_0119 |
|  |  | Alide_0129 | Alide2_0120 |
| Nitrous oxide reductase | Nitrous oxide : dinitrogen gas | Alide_3077 | Alide2_1364 |
|  |  | Alide_3078 | Alide2_1365 |
|  |  | Alide_3079 | Alide2_1366 |
|  |  | Alide_3080 | Alide2_1367 |
|  |  | Alide_3081 | Alide2_1368 |
|  |  | Alide_3082 | Alide2_1369 |
|  |  | Alide_3083 | Alide2_1370 |
