# Supplementary material for: Genome Analysis and Physiological Comparison of Alicycliphilus denitrificans Strains BC and K601T
Source: PLoS One. 2013 Jun 25;8(6):e66971. doi: 10.1371/journal.pone.0066971 (PMC3692508; doi:10.1371/journal.pone.0066971)
Supplement: Table S5 — List of genes involved in degradation of aromatic and acyclic compounds in A. denitrificans strains BC and K601T. (DOCX) [file pone.0066971.s005.docx]

| **Enzyme name** | **Reaction** | **GeneID in BC** | **GeneID in K601^T^** |
| --- | --- | --- | --- |
| Bifunctional hydratase/alcohol dehydrogenase | Cyclohexenone : 1,3-cyclohexanedione | - | Alide2_4320 |
|  |  | - | Alide2_4321 |
|  |  | - | Alide2_4322 |
| Sigma 54 transcriptional regulator |  | Alide_0322 | Alide2_0269 |
| Benzene/phenol-monooxygenase | Benzene/toluene : (methyl-)phenol : (methyl-)catechol | Alide_0323 | Alide2_0270 |
|  |  | Alide_0324 | Alide2_0271 |
|  |  | Alide_0325 | Alide2_0272 |
|  |  | Alide_0326 | Alide2_0273 |
|  |  | Alide_0327 | Alide2_0274 |
|  |  | Alide_0328 | Alide2_0275 |
| Ferredoxin |  | Alide_0329 | Alide2_0276 |
| Catechol 2,3-dioxygenase | (Methyl-)catechol : 2-hydroxymuconic semialdehyde | Alide_0330 | Alide2_0277 |
| Putative regulator of phenolics degradation |  | Alide_0334 | Alide2_0279 |
| 2-Hydroxymuconic semialdehyde dehydrogenase | 2-Hydroxymuconic semialdehyde : 5-hydroxyhexa-2,4-dienedioate | Alide_0335 | Alide2_0280 |
| 2-Hydroxymuconic semialdehyde hydrolase | 2-Methylcatechol : 2-hydroxy-6-oxo-2,4-heptadienoate | Alide_0336 | Alide2_0281 |
| 2-Oxopent-4-enoate hydratase | 2-Oxopentenoate : 4-hydroxy-2-oxopentanoate | Alide_0337 | Alide2_0282 |
| Acetaldehyde dehydrogenase (acetylating) | Acetaldehyde : acetyl-coA | Alide_0338 | Alide2_0283 |
| 4-Hydroxy-2-oxovalerate aldolase | 4-Hydroxy-2-oxovalerate : acetaldehyde | Alide_0339 | Alide2_0284 |
| 4-Oxalocrotonate decarboxylase | 5-Oxohex-2-enedioate : 2-oxopentanoate | Alide_0340 | Alide2_0285 |
| 4-Oxalocrotonate tautomerase family enzyme | 5-Hydroxyhexa-2,4-dienedioate : 5-oxohex-2-enedioate | Alide_0342 | Alide2_0287 |
| Catechol 1,2-dioxygenase | Catechol : *cis,cis*-muconate | Alide_2650 | - |
| Muconate cycloisomerase | *Cis,cis*-muconate : muconolactone | Alide_2651 | - |
| Muconolactone isomerase | Muconolactone : 3-oxoadipate-enol-lactone | Alide_2647 | Alide2_3665 |
| 3-Oxoadipate-enol-lactonase | 3-Oxoadipate-enol-lactone : 3-oxoadipate | Alide_2648 | - |
| 3-Oxoacid CoA transferase | 3-Oxoadipate : 3-oxoadipyl-coA | Alide_3513 | Alide2_3663 |
|  |  | Alide_3514 | Alide2_3664 |
| Acetyl-CoA acetyltransferase | 3-Oxoadipyl-coA : acetyl-coA | Alide_0140 | - |
|  |  | Alide_0678 | - |
|  |  | Alide_1564 | - |
|  |  | Alide_3281 | - |
|  |  | Alide_3761 | - |
